# tDCS peripheral nerve stimulation can enhance passive avoidance learning in rats

**DOI:** 10.3389/fnins.2025.1623434

**Published:** 2025-09-19

**Authors:** Luuk van Boekholdt, Silke Kerstens, Kaydee Decloedt, Myles Mc Laughlin

**Affiliations:** KU Leuven, Department of Neurosciences, Leuven Brain Institute, Leuven, Belgium

**Keywords:** rat, tDCS, transcranial direct current stimulation, peripheral nerve, transcutaneous, passive avoidance task, transcranial

## Abstract

Transcranial direct current stimulation (tDCS) is being considered as a treatment for many psychiatric and neurological disorders. Rodent models of tDCS have been used in several behavioral tasks to demonstrate the technique’s benefits in improving memory and learning. Recent research suggests that peripheral nerve stimulation in tDCS may be responsible for some of its effects. In this work, we first aimed to repeat a previously reported tDCS effect of improved passive avoidance task (PAT) learning in a minimally restrictive rat model and investigate whether peripheral nerve stimulation contributed to these effects by using two additional stimulation groups in which the electric field in the brain (transcranial-only tDCS) and skin (transcutaneous-only tDCS) were separated. Analysis revealed that, at 0.25 mA, none of the stimulation conditions significantly improved PAT learning compared to sham. This non-replication experiment calls for more research to investigate whether tDCS at 0.25 mA can truly improve PAT learning in rats. In a subsequent experiment, we aimed to investigate the effects of transcutaneous-only tDCS at 2 mA, an amplitude more relevant to tDCS in humans. We found that 30 min of DC stimulation at 2 mA, with the cathodal electrode implanted over the third occipital nerve, improved PAT learning. This indicates that DC stimulation of peripheral nerves is capable of modulating learning and memory and supports the theory that peripheral nerve stimulation may contribute to some observed effects of tDCS. More research is necessary to investigate the behavioral and neurophysiological effects of DC peripheral nerve stimulation, and its contribution in tDCS effects observed in animals and humans.

## Introduction

Transcranial direct current stimulation (tDCS) is a popular non-invasive neuromodulation method that is widely used by neuroscientists and clinicians to modulate brain activity and treat a wide range of disorders. Despite the technique’s widespread potential, tDCS suffers from low effect sizes and limited reproducibility across the field. Potential reasons for the low effect sizes and reproducibility include inter-subject variability (Vergallito et al., 2022), low sample size (Minarik et al., 2016) and the lack of active control conditions. Rodent models of tDCS are frequently used to investigate tDCS effects and have shown the technique’s efficacy in healthy rodents in improving memory and learning in several behavioral paradigms including the allothetic place avoidance alternation task (Dockery et al., 2011), Morris water maze (Podda et al., 2016), novel object recognition (Podda et al., 2016) and passive avoidance task (Yu et al., 2019; Jung et al., 2019). These studies administered an anodal or cathodal direct current to the skull, while return electrodes were positioned on the chest or back of the animal using a latex jacket (Dockery et al., 2011), custom corset (Podda et al., 2016) or elastic bandage (Yu et al., 2019; Jung et al., 2019). A downside of these return electrodes is that they require fixation to the rat before stimulation sessions. Moreover, these designs will restrict the free mobility of the animal while stimulation is administered. These stressors could affect subsequent behavior during learning paradigms.

In most experimental designs, sham tDCS acts as a control condition, in which the current is gradually increased in a few seconds up to the target amplitude after which it is ramped down and turned off. While this will, to some extent, mimic the itching and tingling experience of tDCS, higher rates of sensory side effects have been reported in active compared to sham stimulation (Kessler et al., 2012). This raises the question of whether these side effects could have influenced reported results. The itching and tingling experience reported in tDCS evidences the fact that peripheral nerves in the scalp are effectively activated while stimulation is administered. Recent research has suggested that this peripheral nerve activation may not only be a side effect, but could actually be one of the neuromodulatory mechanisms of tDCS (van Boekholdt et al., 2021; Vanneste et al., 2020). One of the nerves that could be stimulated by conventional tDCS montages is the occipital nerve, and recent research suggests a potential role for this nerve in mediating some tDCS learning effects (Luckey et al., 2020; Luckey et al., 2022). Similar to many human tDCS electrode montages, electric fields are also created in both the brain and the skin of many rodent models of tDCS. Therefore, the origin of behavioral effects in these models is not unambiguous. While it has generally been assumed that tDCS behavioral effects in rodents were caused by the electric field in the brain, potential contributions of peripheral nerve stimulation are often not considered, nor controlled for.

One of the most prominent behavioral tDCS effects in rats is an improvement in passive avoidance learning: two research groups have shown that 30 min of anodal DC stimulation on the skull (0.25 mA) can increase memory retention (Yu et al., 2019; Jung et al., 2019). The first work by Yu et al. reported that the enhanced memory performance was accompanied by increased hippocampal CA1 long-term potentiation (LTP) and higher levels of brain derived neurotrophic factor (BDNF) in hippocampal CA1 region in slices from rats that were subjected to the stimulation. Jung et al. repeated this behavioral effect and found that passive avoidance learning was only improved when tDCS was administered before the training session (i.e., the acquisition phase of the memory) and not when it was administered before the testing session (i.e., the retrieval phase of the memory). In addition, this group purified synaptoneurosomes from hippocampi of rats subjected to stimulation and sham. This revealed 184 differentially expressed hippocampal proteins, many of which are associated with receptor signaling and voltage-gated ion channel activity in pathways associated with learning and memory.

These studies thus implicate the hippocampus as a putative mediator of the observed effect on passive avoidance learning. One could be tempted to attribute the observed behavioral effect and hippocampal changes to the electric field created in the hippocampus during DC stimulation. Activation of peripheral nerves could however provide an alternative mode of action. This pathway is also termed the tDCS transcutaneous mechanism (van Boekholdt et al., 2021) and could involve the activation of the ascending reticular activating system (ARAS); a network of ascending tracts that regulate basic behavioral processes, including vigilance and arousal (Kinomura et al., 1996). An important nucleus of the ARAS is the locus coeruleus (LC), which has been shown to be involved in many cognitive processes including attention, learning and memory (Sara, 2009). Interestingly, the LC has been shown to facilitate memory formation through norepinephrinergic (Wagatsuma et al., 2017) and dopaminergic projections to the hippocampus (Kempadoo et al., 2016), posing a putative mechanism through which peripheral nerve stimulation could induce its effects on hippocampal synaptic plasticity, hippocampal protein expression and passive avoidance learning.

Direct evidence that peripheral nerve stimulation can improve passive avoidance learning comes from research stimulating peripheral nerves in an isolated manner in rats: researchers have recently shown that memory retention in the PAT can be improved with vagus nerve stimulation (Olsen et al., 2022) and greater occipital nerve stimulation (Vanneste et al., 2020). Interestingly, this first study also reported that vagus nerve stimulation induced enhanced LTP, spontaneous spike amplitude and frequency and increased BDNF expression in hippocampal CA1. A key difference in the stimulation application in these studies is that researchers implanted cuff electrodes around the nerves and administered pulsed stimulation rather than DC stimulation. It is currently not known whether DC stimulation of peripheral nerves could cause similar behavioral effects and whether it could have contributed to the previously mentioned improved memory outcomes in tDCS rodent models.

In the current study, we first aimed to investigate whether improved passive avoidance task (PAT) memory performance through 0.25 mA tDCS in rats is caused by the transcranial or the transcutaneous mechanism. We specifically assessed passive avoidance task (PAT) memory retention in rats, as memory performance in this paradigm was previously shown to be one of the most prominent behavioral tDCS effects in rats (Yu et al., 2019; Jung et al., 2019). To dissect the transcranial and transcutaneous pathways of tDCS, we used a previously described minimally restrictive rat model of tDCS that allows for the separation of the electric fields in the brain and skin. In a second experiment, we investigated whether PAT memory retention was improved by transcutaneous-only tDCS of 2 mA, a current that more accurately models the transcutaneous component of tDCS in humans. We hypothesized that, at 0.25 mA, either the transcranial or transcutaneous pathway of tDCS would induce improved PAT memory retention. Moreover, we hypothesized that transcutaneous tDCS of 2 mA would induce an improvement in PAT memory retention.

## Materials and methods

### Animals

Male Sprague–Dawley rats of 5–8 weeks old (*n* = 16 per stimulation group for experiment 1; *n* = 13 per stimulation group for experiment 2) were obtained from Charles Rivers Laboratory. Rats had ad libitum access to food and water and were housed at 19°C on a 14/10 h light/dark cycle (light off at 9:00 P. M.). Experiments were approved by the KU Leuven ethics committee (project 072/2020 and 074/2022).

### Experimental procedure

Distinct cohorts of rats were used for experiments 1 and 2. Rats used in experiment 1 were implanted with epicranial and subcutaneous electrodes, while rats used in experiment 2 were only implanted with subcutaneous electrodes. In experiment 1, rats were randomly divided into four stimulation groups: regular tDCS, transcutaneous-only tDCS, transcranial-only tDCS and sham. Rats in experiment 2 were randomly divided into two stimulation groups: transcutaneous-only tDCS and sham. Seven days after the surgical implantations, electrical or sham was administered for 30 min through electrode combinations corresponding to the animal’s stimulation group (see below). Immediately after the stimulation, the training (encoding) session was performed in the PAT setup. The testing (retrieval) session was conducted 24 h later. The whole experimental procedure, including surgery, animal handling and behavioral testing, was performed by an experimenter that was blinded to the stimulation group. During the 30 min of electrical stimulation, a second experimenter administered electrical or sham stimulation through electrode combinations corresponding to the animal’s stimulation group. The experimental group could not be reliably guessed by the blinded experimenter, as previously established in this rat model (van Boekholdt et al., 2023). Further details of the procedures are provided in the corresponding sections below.

### Surgery preparation

Rats weighed 300–400 grams at the time of surgery and were anesthetized with the following mixture: 3X ketamine (100 mg/mL Nimatek): 2X medetomidine (1 mg/mL Domitor): 5X saline, adapted from established protocols for rat anesthesia (Jang et al., 2009). After an initial i.p. injection (100 μL/100 g bodyweight), anesthesia depth was routinely monitored with the toe-pinch reflex and additional anesthesia was administered (i.p., 25 μL/100 g bodyweight) when anesthesia depth was low. The anesthetized animal was fixated in a stereotactic frame and a heating pad and rectal probe thermometer were used to maintain the body temperature of the rats.

### Epicranial electrode implantation (experiment 1)

The scalp of the rat was shaved and the skull was exposed and cleaned with 1.2% hydrogen peroxide. For epicranial stimulation electrodes, a conductive paste (Ten20) was applied underneath and inside an M2 nut (Farnell M2- HFA2-S100) and a brass ring (outer ⌀ = 6 mm, inner ⌀ = 1.7 mm) so that the entire outer surface area (including the holes) could be used as stimulation surface. Both electrodes were positioned on the midline: the M2 nut 2.5 mm caudal to bregma and the brass ring 3 mm caudal to lambda. Four bone anchor screws were drilled into the skull and fixed to the epicranial stimulation electrodes with dental cement to keep them in place. Small screws attached to socket contacts (Bilaney #E363/20/1.6) were inserted in the electrode holes (without penetrating the skull) to connect them to an electrode pedestal (Bilaney #MS363). The pedestal was fixed to the skull and bone anchor screws with dental cement and the skin was sutured around the protruding electrode pedestal.

### Subcutaneous electrode implantation (experiments 1 and 2)

The rat was shaved on the left dorsal neck area over the third occipital nerve and central back area (centered over the spine). Skin pockets were created in the shaved areas and circular disk electrodes of conductive rubber (Neurocare group #305090–01; ⌀ = 10 mm) were implanted subcutaneously. The hollow rod extending from the center of the electrodes was left to protrude from the skin. Surgical glue (Vetbond) was used to close the skin pockets.

Note: extensive step-by-step documentation with graphical illustrations of the epicranial and subcutaneous electrode implantations can be found in previously published work (van Boekholdt et al., 2023).

### Post-surgery care and handling

Antibiotic cream (Fucidin) was applied to the surgical wounds and general analgesic (Metacam, 1 mg/kg bodyweight) was injected subcutaneously after the surgery. Rats were housed individually after the surgery to improve recovery and reduce inter-subject variability. All rats were allowed exactly 7 days of recovery before being used in experimentation. On all weekdays post-surgery, except the day after the surgery, rats were handled extensively to minimize handling-related stress during experimentation.

### Stimulation groups (experiment 1)

**Figure 1:**
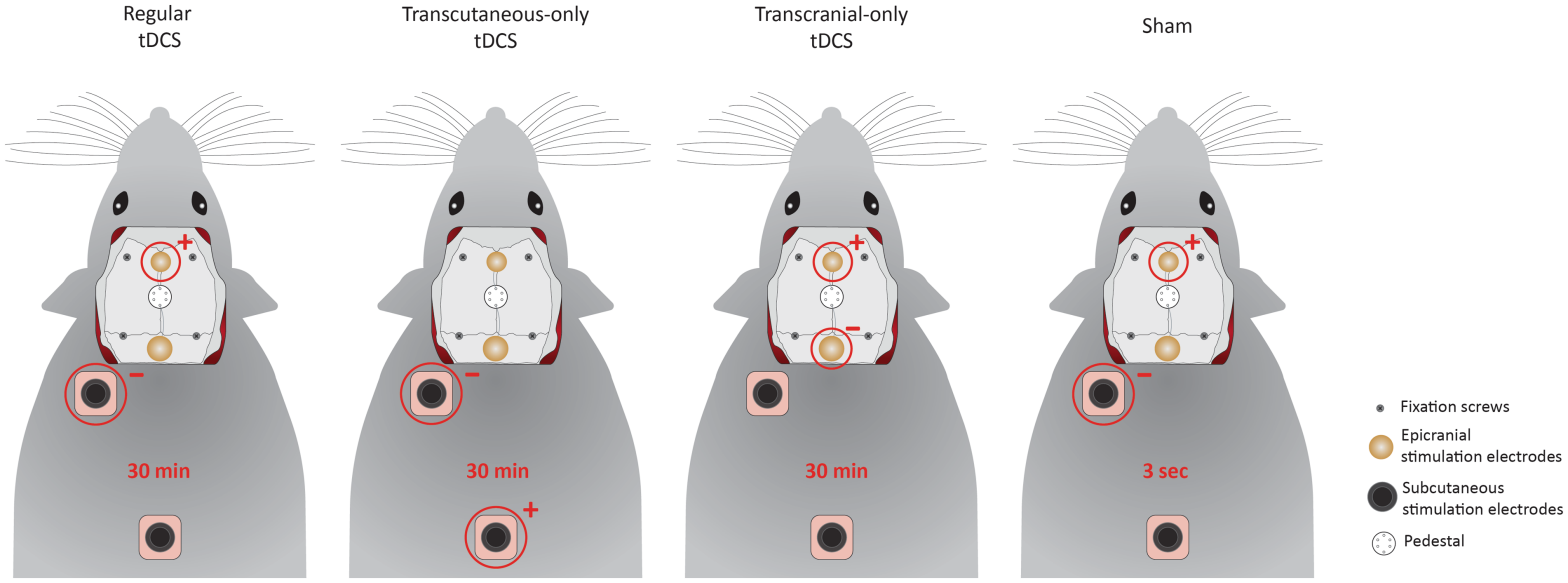
Stimulation groups in experiment 1. The electrodes used for different stimulation groups are encircled in red and polarity is indicated with a plus-sign for anodal and a minus-sign for cathodal electrodes. In regular tDCS, the ventral epicranial electrode and subcutaneous electrode over the third occipital nerve were used for stimulation. Only subcutaneous electrodes were used in transcutaneous-only tDCS while transcranial-only tDCS was performed through the epicranial electrodes. Sham was performed by turning on stimulation through the regular tDCS electrodes for the first 3 s.

Rats were randomly divided into the following stimulation groups: regular tDCS, transcutaneous-only tDCS, transcranial-only tDSC, and sham (Figure 1). In regular tDCS, stimulation was performed through the ventral epicranial electrode (anodal) and subcutaneous electrode over the third occipital nerve (cathodal). For transcutaneous-only tDCS (i.e., stimulation of the skin, but not the brain), the anodal electrode was changed to the subcutaneous electrode on the central back. For transcranial-only tDCS (i.e., stimulation of the brain, but not the skin), the ventral (anodal) and dorsal (cathodal) epicranial electrodes were used. In the sham group, stimulation was turned on for only 3 s through the regular tDCS electrode combination, but cables remained connected throughout the full 30 min.

### Stimulation groups (experiment 2)

Rats, which were only implanted with the subcutaneous electrodes, were randomly divided into the following groups: transcutaneous-only tDCS and sham. The locations of the electrodes were the same as the locations in experiment 1. In sham condition, stimulation was turned on for only 3 s through the subcutaneous-only electrode combination, but cables remained connected throughout the full 30 min.

### Electrical stimulation (experiment 1)

Seven days post-surgery, the rats were moved, in their home cage, to the experiment room. While rats remained in their home cage, a connector cable with a 363 plug (Bilaney) was used to connect the electrode pedestal on the rat’s head to a swivel system (Bilaney #SL12C). Wired crocodile clips (Mueller BU-30) soldered onto a 363 plug (Bilaney) were clipped onto the subcutaneous electrodes and connected to the swivel. All electrodes were connected by the experimenter that was blinded to the stimulation group. At approximately 9:30 a.m., a second experimenter administered 30 min of 0.25 mA electrical (or sham) stimulation using a current stimulator (A-M Systems, Model 2,200) through two electrodes, corresponding to the rat’s stimulation group. During these 30 min, rats were allowed to freely roam around their home cage. When rats attempted to climb the sides of the cage they were gently nudged back by the blinded observer. After stimulation, the stimulation cables were disconnected from the rat.

### Electrical stimulation (experiment 2)

In these rats, only subcutaneous electrodes were implanted. The subcutaneous electrodes were connected to the swivel. At approximately 2:00 p.m., rats received 30 min of 2 mA subcutaneous-only tDCS or sham. The rest of the electrical stimulation was the same as in experiment 1.

### Passive avoidance task

The training session of the PAT was carried out immediately after electrical stimulation in an automated passive avoidance system (Ugo Basile, Italy). During this session, rats were placed in the illuminated (safe) compartment. After 30 s, the door connected to the dark compartment was automatically opened. When rats crossed to enter the dark compartment, the door automatically closed, and a foot shock (0.8 mA for 1 s) was administered. Rats remained in the dark compartment for an additional 30 s before they were returned to their home cage. Approximately 24 h later, the testing session was performed, in which rats were returned to the illuminated compartment. After 30 s, the door was automatically opened and the step-through latency to enter the dark compartment was measured. The cut-off value was set at 600 s, meaning that a step-through latency of 600 s was registered when rats remained in the illuminated compartment for 600 s.

### Statistics

GraphPad Prism was used for analyzing and visualizing data. Data were tested for normality via the Shapiro–Wilk test. As several variables were not normally distributed, nonparametric tests were applied. Inter-trial differences (training session vs. testing session) within groups were analyzed with the two-tailed Wilcoxon matched-pairs signed rank test, as the data consisted of paired observations. Between-group differences in the first experiment (sham vs. regular tDCS vs. transcranial-only tDCS vs. transcutaneous-only tDCS) were analyzed with the Kruskal-Wallis test, because there were more than two independent groups. The two-tailed Mann–Whitney U test was used to analyze between-group differences in the second experiment (sham vs. transcutaneous-only tDCS), because there were two independent groups. Differences were considered as significant at *p* < 0.05. The sample size was determined by performing a power analysis using the estimated effect sizes of PAT studies with a similar experimental design (Yu et al., 2019; Jung et al., 2019) with *α* = 0.05 and power (1–*β*) = 0.8. Experiment 2 included fewer animals than experiment 1 because the comparison involved only two groups, and the Mann–Whitney U test provides greater statistical power than the four-group Kruskal–Wallis test.

## Results

### Experiment 1: effects of 0.25 mA DC stimulation on PAT learning

This experiment was designed to investigate whether improved PAT performance by tDCS could be replicated in a minimally invasive rat model and assess whether the effect was caused by the electric field in the brain (i.e., the transcranial mechanism) or by the electric field in the skin (i.e., the transcutaneous mechanism). To answer both these questions, the PAT was performed in four groups of rats with different stimulation conditions: ‘regular tDCS’, ‘transcutaneous-only tDCS’, ‘transcranial-only tDCS’, and ‘sham’.

Within-group analyses were performed using the Wilxocon matched-pairs signed rank test and revealed that rats in all groups had a greater step-through latency in the testing session compared to the training session, indicating that learning was successful in all groups. For sham, Wilcoxon matched-pairs signed rank (*n* = 16), W = 112.0, *p* = 0.0021, median of differences = 57.50. For regular tDCS, Wilcoxon matched-pairs signed rank (*n* = 16), W = 120.0, *p* = 0.0008, median of differences = 80.10. For transcutaneous-only tDCS, Wilcoxon matched-pairs signed rank (*n* = 16), W = 120.0, p = 0.0008, median of differences = 76.80. For transcutaneous-only tDCS, Wilcoxon matched-pairs signed rank (*n* = 16), W = 124.0, *p* = 0.0004, median of differences = 57.10.

**Figure 2:**
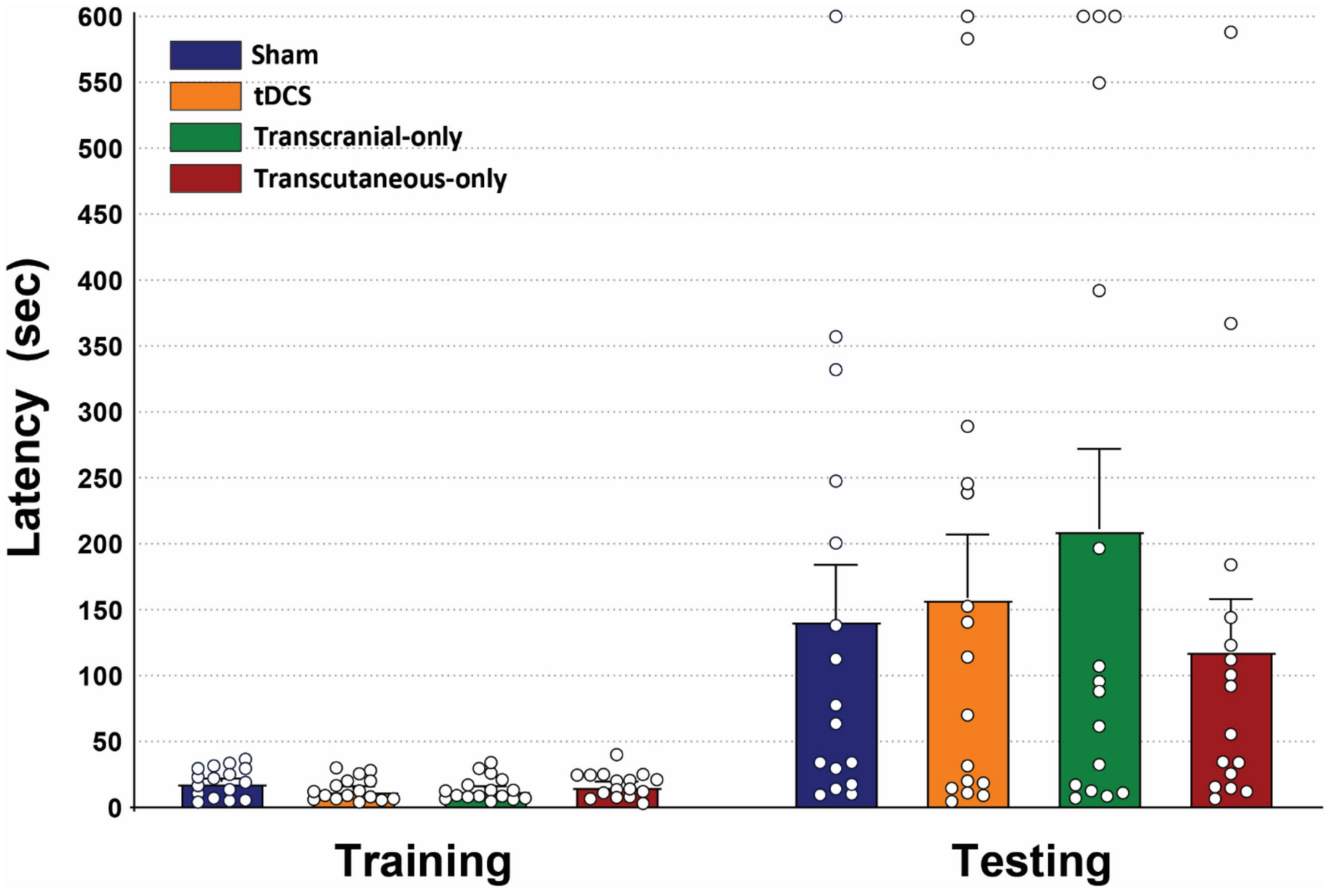
Thirty minutes of DC stimulation at 0.25 mA did not significantly alter memory retention in the passive avoidance task in any of the stimulation groups. Kruskal-Wallis test showed no significant differences in the step-through latencies between any of the groups during training and testing sessions. Data are presented as mean ± SEM; *N* = 16 rats per group.

Between-group analysis showed no initial differences in step-through latency between groups during the training session (Kruskal-Wallis; *n* = 16 per group, total *n* = 64); H(3) = 3.071, *p* = 0.3809, suggesting that the different stimulation conditions did not significantly influence initial exploratory behavior during the training session. Analysis of the testing session showed no differences in step-through latency between any of the groups (Kruskal-Wallis; *n* = 16 per group, total *n* = 64); H(3) = 0.264, *p* = 0.9666, indicating that none of the administered stimulation conditions significantly altered memory retention compared to sham. This means, firstly, that the effect of improved memory retention in the PAT through 30 min of tDCS (0.25 mA) reported by other groups could not be replicated in our minimally restrictive rat model. Secondly, we were not able to test the hypothesis that effects were caused by either the transcranial or the transcutaneous mechanism (Figure 2).

### Experiment 2: effects of 2 mA transcutaneous-only stimulation on PAT learning

The administered current of 0.25 mA, used in experiment 1 is lower than the typical 1–2 mA used in human tDCS protocols to account for the higher portion of the electric field reaching the brain in rats compared to human tDCS. Importantly however, this entails that the applied current through the subcutaneous electrodes is also decreased to 0.25 mA. This motived us to explore how PAT learning is affected by transcutaneous tDCS with amplitudes more closely resembling human tDCS. In a follow-up experiment we therefore investigated the effect of 2 mA DC stimulation through the subcutaneous electrodes. Rats were only implanted with the subcutaneous electrodes and the PAT was performed with two stimulation groups: a ‘transcutaneous-only tDCS’ group and a sham group.

Within-group analysis showed that step-through latency in the testing session was greater than in the training session, indicating that both groups learned the task successfully. For sham, Wilcoxon matched-pairs signed rank (*n* = 13), W = 75.00, *p* = 0.0061, median of differences = 61.40. For transcutaneous-only tDCS, Wilcoxon matched-pairs signed rank (*n* = 13), W = 85.00, *p* = 0.0012, median of differences = 282.1.

**Figure 3:**
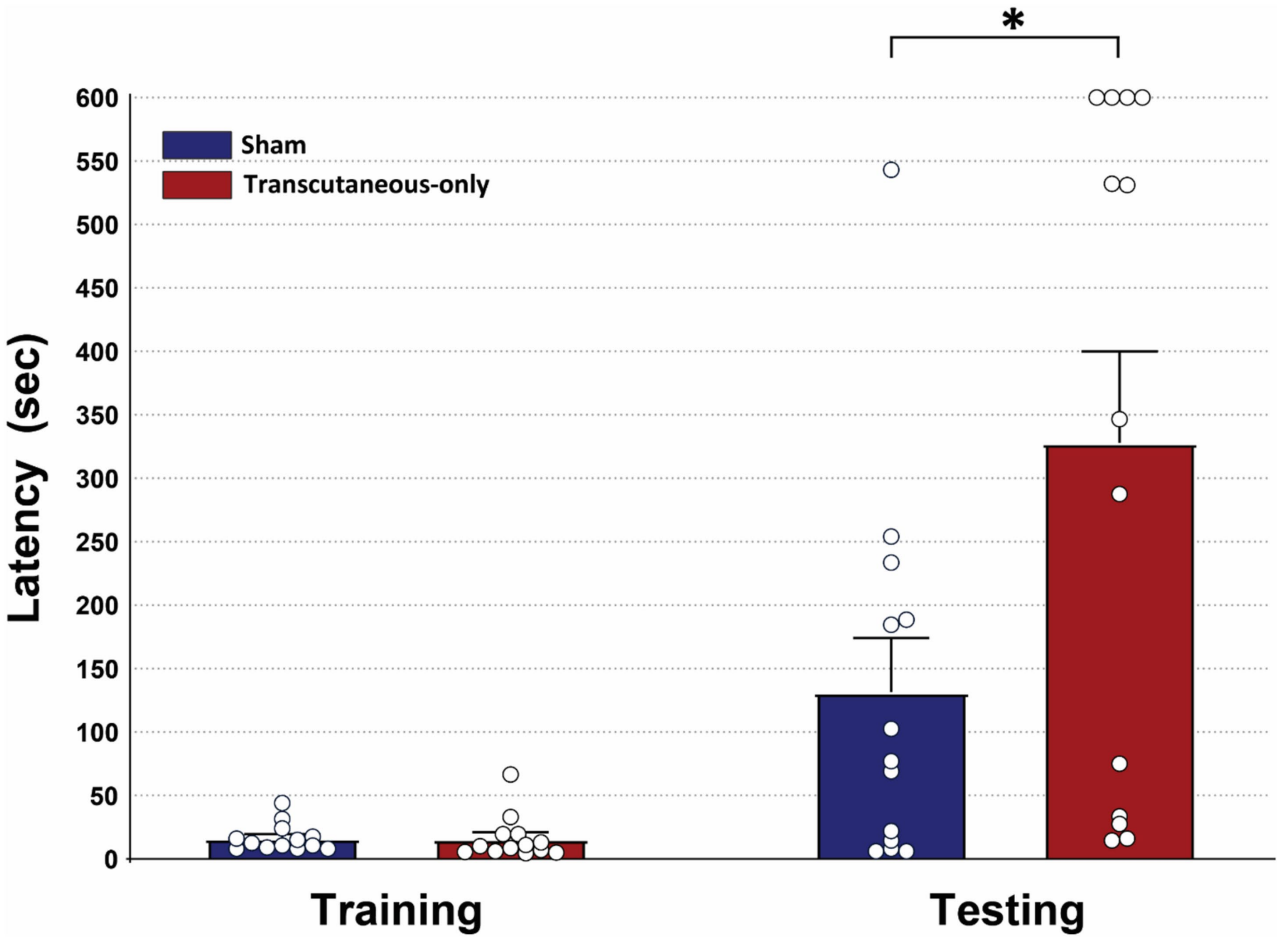
Memory retention in the passive avoidance task was improved by 30 min of transcutaneous-only DC stimulation at 2 mA. Mann–Whitney U tests of the step-through latencies showed no significant difference between sham and transcutaneous-only tDCS during the training session, but found a significant difference between sham and transcutaneous-only tDCS during the testing session. Data are presented as mean ± SEM; *N* = 13 rats per group; *p < 0.05.

Between-group analysis revealed that initial step-through latency was similar during the training session (Mann–Whitney; *n* = 13 per group, total *n* = 26; U = 65, *p* = 0.3291, difference between medians = −2.2), suggesting that initial exploratory behavior was not significantly influenced by the stimulation. Analysis of the testing session revealed a significantly higher step-through latency in the transcutaneous-only tDCS group compared to the sham group (Mann–Whitney; *n* = 13 per group, total *n* = 26; U = 46, *p* = 0.0480, difference between medians = 269.4), showing that memory retention was significantly increased in the transcutaneous-only tDCS group. This indicates that 30 min of DC stimulation at 2 mA through subcutaneous electrodes can improve memory retention in the PAT in rats (Figure 3).

Group summary statistics (mean ± SD) for experiment 1 and 2 are provided in Supplementary Table 1.

## Discussion

### No replication of improved PAT memory retention after 0.25 mA tDCS

In the first experiment, we did not observe increased PAT learning in any of our stimulation groups compared to sham. This means that, somewhat surprisingly, we could not replicate the previously reported (Yu et al., 2019; Jung et al., 2019) improvement of PAT learning with 30 min of 0.25 mA tDCS (i.e., no improvement in our tDCS group vs. sham). Secondly, we were unable to assess whether potential PAT improvements were caused by the electric field in the brain or in the skin (i.e., no improvement in our transcranial- or transcutaneous-only tDCS vs. sham).

### Differences with previous studies

We analyzed differences in experimental design between our first experiment and the experiments of previous studies (Yu et al., 2019; Jung et al., 2019) that could explain the lack of behavioral effect observed in our experiment. The biggest difference is the peripheral reference electrode. In the previous studies, a cutaneous electroencephalography electrode with a diameter of 10 mm (Yu et al., 2019)^,^ or 12 mm (Jung et al., 2019) was attached to the anterior chest using elastic bandages. In contrast, we used a subcutaneous electrode with a diameter of 10 mm implanted over the left 3^rd^ occipital nerve (García-Magro et al., 2018). This poster location was chosen to allow us, in contrast the anterior chest, to perform chronic implantation and stimulation with the subcutaneous electrode. If improved passive avoidance learning in the previous rat experiments was caused by the transcutaneous mechanism, these peripheral electrode differences could have caused us to miss the effect. It is possible that peripheral nerves in the anterior chest were responsible and necessary for the behavioral effect. Notably, the vagus nerve innervates many organs in the anterior trunk (Baquiran, 2022), and 30 mins of vagus nerve stimulation was recently shown to improve passive avoidance learning in rats (Olsen et al., 2022). It is unclear however, whether the anterior chest electrodes in the other studies could have stimulated the vagus nerve in the anterior trunk. Another possibility is that by using subcutaneous instead of cutaneous electrodes, we stimulated peripheral nerves less efficiently, possibly due to differences in cutaneous and subcutaneous nerve fibers availability and sensitivity. It is also possible that the current through the subcutaneous electrodes was buffered by body fluids, reducing the activation of peripheral nerves.

Another difference between our study and the previous studies is the anodal epicranial electrode. Although the location of this electrode was identical, we used the full surface area (including the hole) of a hexagonal M2 nut (13.86 mm^2^ based on the short diagonal of 4 mm), while previous studies used a circular electrode (19.64 mm^2^). The smaller surface area, and consequently higher current density (18.04 A/m^2^ for our electrode, 12.73 A/m^2^ for the circular electrode), likely induced a stronger but spatially narrower electric field in the brain. Thus, if improved passive avoidance learning in the previous experiments was caused by the transcranial mechanism, we may have missed the effect due to the difference in epicranial electrode. Finally, our study differs in the rat model being minimally restrictive while the rat models used previously limited (to some extent) the mobility of the rats during tDCS application due to the elastic bandage wrapped around the animals. In theory, this limited mobility could be a necessary factor to induce improved memory retention, but we deem this unlikely.

### No PAT learning effects after transcranial- and transcutaneous-only tDCS

The transcranial- and transcutaneous-only tDCS conditions were added to investigate whether potential effects in the tDCS group were caused by the epicranial (anodal) electrode or the subcutaneous (cathodal) electrode. The conditions were therefore designed to separate the electric fields in the brain and skin, while minimizing additional effects of the return electrodes. In the transcranial-only condition this was done by using a large return (cathodal) electrode (⌀ = 6 mm; area = 56.55 mm^2^) with a current density of 4.42 A/m^2^, compared to a current density of 18.04 A/m^2^ of the anodal electrode. In the transcutaneous-only condition, the cathodal (and presumably active) electrode was positioned over the 3^rd^ occipital nerve (as in the regular tDCS condition). The subcutaneous (anodal) return electrode was positioned over the central back of the animal; an area with a relatively low innervation density (Corniani and Saal, 2020) and further away from cranial nerves (Rea, 2014).

Given that the return electrodes were designed to be minimally effective, and no altered memory retention was observed in the tDCS group, it is not surprising that transcranial- and transcutaneous-only tDCS did not induce a significant effect on memory retention either. There was however a notable, but non-significant increase in the step-through latency in the transcranial-only tDCS compared to the sham group. If this increase was caused by the stimulation, we can probably attribute it to the transcranial mechanism, as only epicranial electrodes were used in this condition. Interestingly, while not significant, the transcutaneous-only group showed the lowest step-through latency of all groups, and the tDCS-group (with both transcranial and transcutaneous tDCS) showed a lower step-through latency than the transcranial-only group (without transcutaneous tDCS). If this trend reflects a true effect, it may suggest that the transcutaneous-only component might decrease memory retention at this stimulation amplitude.

### Implications of non-replication

The lack of significant increase in PAT learning in the tDCS and transcranial-only groups challenges the previously presumed notion that tDCS over the skull of the rat can increase passive avoidance learning, especially given the substantial sample size of 16 rats per group. However, it is possible that one of the previously described differences in the intervention method may have caused the failure to replicate the earlier findings. This is a limitation of the current study. More research is necessary to show whether tDCS in rats can truly improve PAT learning at 0.25 mA and separation of the transcranial and transcutaneous modalities of tDCS can give us insights into the mechanisms underlying observed effects.

### Improved passive avoidance learning after 2 mA transcutaneous-only tDCS

In the second experiment, we showed that 2 mA DC stimulation through the subcutaneous electrodes, administered for 30 min prior to the training session, improved memory retention in the PAT. We used a stimulation amplitude of 2 mA to more closely match the stimulation amplitude levels typically used in human tDCS protocols. However, since the electrodes in the rats are implanted subcutaneously instead of cutaneously, the exact degree of peripheral nerve activation compared to human tDCS is not straightforward to determine. Differences between cutaneous and subcutaneous nerve fiber availability and sensitivity could cause differential activation of peripheral nerves in human tDCS compared to our subcutaneous electrodes. It should also be noted that the size of the subcutaneous stimulation electrode is smaller than that of the typical electrode used in human tDCS. This will result in a higher current density at the electrode-tissue interface which could result in a higher activation of peripheral nerves. However, we also expect that highly conductive body fluids immersing the subcutaneous electrodes could act as a buffer, reducing peripheral nerve stimulation. Altogether, we expect that there was no excessive peripheral nerve activation in our experiment. This is evidenced by results from previous work in the rat model (van Boekholdt et al., 2023), in which blinded observers could not correctly predict whether rats received 2 mA stimulation through the subcutaneous electrodes implanted at the same locations, suggesting a lack of strong behavioral responses and thus suggesting no excessive peripheral nerve activation. In contrast, human subjects do report sensory side effects such as tingling and itching. We therefore expect that peripheral nerve activation with our subcutaneous electrodes at 2 mA DC was not disproportionally larger than peripheral nerve activation in human tDCS experiments.

### Implications

This is, to our knowledge, the first animal experiment that shows that peripheral nerve stimulation with a DC current can increase memory retention. We think that this effect was most likely induced by the cathodal electrode over the 3^rd^ occipital nerve. The occipital nerve is a cranial nerve that has been posed as potential mediator of some tDCS effects (Luckey et al., 2022; Luckey et al., 2020), possibly through locus coeruleus activation by the ARAS system (van Boekholdt et al., 2021). This result furthermore adds to the body of evidence showing that electrical stimulation of peripheral nerves can improve PAT learning (Vanneste et al., 2020; Olsen et al., 2022). An important difference in the current study however, is the use of a constant current instead of pulsed stimulation through cuff electrodes. The stimulation method is therefore more similar to human tDCS. This result has important implications for the tDCS field and calls for reevaluation of effects in human subjects. As peripheral nerves are also stimulated in human tDCS, as evidenced by the technique’s sensory side effects, it is possible that peripheral nerve stimulation (i.e., the transcutaneous mechanism) could have contributed to behavioral effects in human subjects previously attributed to the electric field in the brain (i.e., the transcranial mechanism). We recommend further implementation of control conditions in tDCS experimental design to investigate the contribution of peripheral nerve stimulation. In animal experiments, this is possible with stimulation conditions that stimulate peripheral nerves without causing an electric field in the brain, as we have done in this work. In human tDCS experiments, this is possible by using control conditions in which topical anesthetics silence peripheral nerves (Kerstens et al., 2022). A clearer understanding of the neurophysiological mechanisms underlying tDCS effects will allow us to deliberately direct research and resources toward more effective tDCS stimulation approaches and further advance the field.

## Data Availability

The raw data supporting the conclusions of this article will be made available by the authors, without undue reservation.
