# Supplementary material for: tDCS peripheral nerve stimulation can enhance passive avoidance learning in rats
Source: Front Neurosci. 2025 Sep 19;19:1623434. doi: 10.3389/fnins.2025.1623434 (PMC12491212; doi:10.3389/fnins.2025.1623434)
Supplement: Supplementary file 1 [file Table_1.DOCX]

|  | **Group** | **Sample size** | **Training session (mean ± SD)** | **Testing session (mean ± SD)** |
| --- | --- | --- | --- | --- |
| **Experiment 1** | Sham | 16 | 19.73 ± 11.54 | 142.53 ± 177.8 |
|  | Regular tDCS | 16 | 13.89 ± 8.65 | 159.14 ± 207.6 |
|  | Transcranial-only tDCS | 16 | 14.19 ± 9.04 | 211.34 ±269.6 |
|  | Transcutaneous-only tDCS | 16 | 17.46 ± 9.48 | 119.53 ± 155.0 |
| **Experiment 2** | Sham | 13 | 16.64 ± 10.83 | 131.63 ± 152.99 |
|  | Transcutaneous-only tDCS | 13 | 16.32 ± 17.18 | 328.12 ± 260.91 |

**Supplementary Table 1.** Mean ± SD values for experiment 1 and 2.
